# Synergistic Interaction between Selective Drugs in Cell Populations Models

**DOI:** 10.1371/journal.pone.0117558

**Published:** 2015-02-11

**Authors:** Victoria Doldán-Martelli, David G. Míguez

**Affiliations:** Departamento de Física de la Materia Condensada, Condensed Matter Physics Center (IFIMAC) and Instituto Nicolás Cabrera, Facultad de Ciencias, Universidad Autónoma de Madrid, Madrid, Spain; Universitat Pompeu Fabra, SPAIN

## Abstract

The design of selective drugs and combinatorial drug treatments are two of the main focuses in modern pharmacology. In this study we use a mathematical model of chimeric ligand-receptor interaction to show that the combination of selective drugs is synergistic in nature, providing a way to gain optimal selective potential at reduced doses compared to the same drugs when applied individually. We use a cell population model of proliferating cells expressing two different amounts of a target protein to show that both selectivity and synergism are robust against variability and heritability in the cell population. The reduction in the total drug administered due to the synergistic performance of the selective drugs can potentially result in reduced toxicity and off-target interactions, providing a mechanism to improve the treatment of cell-based diseases caused by aberrant gene overexpression, such as cancer and diabetes.

## Introduction

The field of modern pharmacology aims to develop novel approaches to improve disease treatment, reduce side effects, minimize costs and enhance the efficiency of targeted therapy. These major challenges require a rational design of novel drugs and improved treatment strategies. In this direction, two of the main approaches currently being pursued involve the development of selective drugs and the design of optimal drug combination therapies.

Drug selectivity can be defined as the ability of a compound to exhibit enhanced effect towards a particular cell population in preference to others. To achieve that, a drug must be designed to target specific cellular components that are differentially expressed in two cell types. In the context of diseases that involve cells overexpressing certain genes, such as oncogenes in cancer [1], this targeting potential can be used to selectively affect only cells with increased levels of the overexpressed protein. Once selectivity is achieved, the drug can be designed to either restore normal cellular function when possible, or to trigger apoptosis of the unhealthy cells without harming the healthy cellular environment. In general, selective drugs are composed of a targeting element (TE) that recognizes and binds to the target protein, and an activity element (AE) that is directed towards the selectively targeted cells. Many of these synthetic chimeric compounds have shown good *in vivo* performance, and several of them have been approved by the FDA or currently undergoing clinical trials [2–15].

On the other hand, drug combination therapies have shown enhanced efficiency compared to individual drug therapy in many diseases [16], including cancer [17, 18] and HIV’s [19]. The interaction between drugs in multicomponent therapies is a complex and multi-scale problem [20] that requires full characterization of the direct and indirect molecular aspects of the interaction, which are often unknown. Due to this, experimental studies and discoveries of successful drug combinations are often based on empirical intuition and trial-and-error approaches. In general, drug interactions can be classified based on their effect when combined, compared to their effect when applied alone. Drugs that do not interact with each other, or are mutually exclusive by competing for the same target are considered as additive [22]. This basically means that the lower concentration which produces a certain effect corresponds to the most potent drug, and there is no gain due to the combination of the two drugs. On the other hand, antagonism occurs when one of the drugs mitigates or counteracts the action of the other, i.e, the combination is always less effective than the single agents at the same concentration. Finally, synergism occurs when the combination of both drugs is more effective than each agent separately at the same total concentration, i.e., one of the agents enhances the actions of the other [21]. This can occur either via direct interaction, i.e, one drug increases the bioavailability of the other, or indirectly, i.e, the two drugs cooperate on targets on the same or different pathways involved in the same process [23]. Thus, the total concentration of drug administered to achieve a certain effect is reduced, which potentially also reduces side effects, drug resistance and undesired off-target interactions.

In the context of selective drugs, synergism and antagonism can be also defined in terms of the enhanced or reduced selective potential of the two drugs when combined [24], i.e, their ability to target selectively a specific cell population, compared to their selective potential when applied individually. In this way, two drugs are synergistic if their combination is more selective than the two drugs acting alone at the same total concentration. Here, we explore the mechanism of interaction between selective drugs in combination from a theoretical perspective. To do that, we develop a population model where two sets of cells expressing different levels of a target molecule are treated with different concentrations of two drugs simultaneously. In principle, these two drugs can be monomeric non-selective ligands (i.e., they do not differentiate between healthy and unhealthy cells), or chimeric ligands, composed of an AE and linked to a TE, allowing them to selectively target unhealthy cells, leaving the healthy environment undamaged.

Two different approaches are taken into account: first, we analyze the effect of combinations of two different chimeric ligands when applied simultaneously to an heterogeneous population of cells; next, we combine the effect of individual chimeric drugs based on the Loewe additivity model [22]. Both models predict that drug combination of selective drugs is synergistic in terms of their selective potential. Finally, we introduce phenotypic inheritance in the cell population to show that both selectivity and synergism also occur in a context where the amount of target proteins of the daughter cells depends on the mother cell, such as in diseases caused by mutations in specific genes. Our results show that the concentration to obtain a desired selectivity can be minimized by simultaneous treatment of selective drugs.

## Models

**Fig 1:**
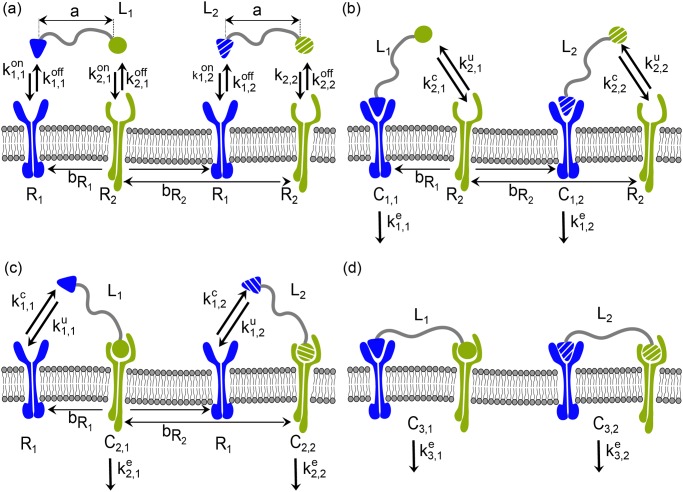
Scheme of the model for the simultaneous interaction of two chimeric ligands with their corresponding receptors. (A) Each one of the ligands *L*_*j*_ consists of two subunits which can interact with their corresponding receptors to form intermediate complexes *C*_*i*,*j*_. (B,C) This first binding event induces an increase in the local concentration of the free subunit of the ligand facilitating the interaction with its corresponding receptor to form complex *C*_3,*j*_ (D).

To analyze the effect of selective drug combinations in a multicellular approach, we develop a mathematical framework where we allow two asynchronous populations of cells with two distinct average number of target molecules to proliferate for a given time. Cells are treated with different concentrations of monomers and chimeric drugs alone or in combination, to then monitor and compare the dynamics of proliferation of the two cell populations. The dynamics of the effect of chimeric drugs at the cellular level is calculated based on a chemical kinetics model for the ligand-receptor interaction [25]. The model used is an extension of our previous contribution to the study of the dynamics of chimeric ligands, where we develop a mathematical framework to predict the selective potential of chimeric drugs, based on the affinity of both AE and TE subunits of the ligand towards their targets (activity element receptor, AER, and targeting element receptor, TER, respectively), the concentration of the target molecules and the linker length between AE and TE in the chimera [25]. The model is rewritten to take into account the simultaneous interaction of two chimeric ligands, resulting in the following set of interactions:

$$\begin{array}{c} R_{i}+L_{j}\;\overset{k_{i,j}^{on}}{\underset{k_{i,j}^{off}}{⇌}}\;C_{i,j} \end{array}$$ (1)

$$\begin{array}{c} C_{1,j}+R_{2}\;\overset{k_{2,j}^{c}}{\underset{k_{2,j}^{u}}{⇌}}\;C_{3,j} \end{array}$$ (2)

$$\begin{array}{c} C_{2,j}+R_{1}\;\overset{k_{1,j}^{c}}{\underset{k_{1,j}^{u}}{⇌}}\;C_{3,j} \end{array}$$ (3)

$$\begin{array}{c} C_{i,j},C_{3,j}\;\overset{k_{i,j}^{e},k_{3,j}^{e}}{\to }\;∄ \end{array}$$ (4)

where *R*_*i*_ corresponds to the *i* receptor (*i* = 1,2) and *L*_*j*_ corresponds to each of the two different ligands (*j* = 1,2) used in the combined treatment. Each ligand *L*_*j*_ is composed of a AE and TE, and it can bind to *R*_1_ or *R*_2_ via reaction 1 to give an intermediate complex *C*_*i*,*j*_ (Fig. 1A–C). These intermediate complexes facilitate reactions 2 and 3 by originating a local concentration of the free subunit of the ligand *L*_*j*_ in the vicinity of the complementary receptor, to generate the complex *C*_3,*j*_ (Fig. 1D). The coupling ($k_{i,j}^{c}$) rate constants in reaction 2 and 3 are calculated as follows:

$$k_{i,j}^{c}={\left(\frac{1}{k_{i}^{diff}}+\frac{1}{k_{i,j}^{on^{\prime }}}\right)}^{-1}$$ (5)

where the diffusive rate constant $k_{i}^{diff}$ is modulated by the diffusion *D*_*i*_ of the receptors *R*_*i*_ at the membrane as [26, 27]:

$$\begin{array}{c} k_{i}^{diff}=\frac{2\pi \;(\sum D_{i})}{A\cdot \log(b_{i}/a)} \end{array}$$ (6)

being *A* the average cell surface area, $b_{i}=\sqrt{A/(\pi R_{i})}$ corresponds to half the average distance between *R*_1_ and *R*_2_, and *a* is the linker length. Effective affinity and dissociation rates for the reactions that take place at the membrane are calculated as [26]:

$$\begin{array}{c} k_{i,j}^{on^{'}}=\frac{k_{i,j}^{on}}{N_{av}\cdot V_{i}} \end{array}$$ (7)

$$\begin{array}{c} k_{i,j}^{u}=\left(1-\gamma_{i,j}\right)k_{i,j}^{off} \end{array}$$ (8)

where *N*_*av*_ is Avogadro’s number and *V*_*i*_ = *A* ⋅ (*h*_*i*_ + *a*) is the effective reaction volume for the second binding event, assumed as a spherical gasket above the cell surface where the free subunit gets distributed after the first binding event (see [25]), being *h*_*i*_ the height of *R*_*i*_ above the cell surface. $\gamma_{i,j}=k_{i,j}^{on\prime }/(k_{i}^{diff}+k_{i,j}^{on\prime })$ corresponds to the capture probability factor for receptor *R*_*i*_ and ligand *L*_*j*_, explained in detail in [26].

For a given constant concentration of both ligands *L*_*j*_, the equations are solved for each individual cell in the population, based on its amount of *R*_1_ and *R*_2_ receptors, identified as TER and AER, respectively. The maximum value of AER-AE complexes formed in each cell is then correlated with the physiological response produced by the AE using experimental dose-response curves (this correlation is a multi-step process explained in detail in Ref. [25]). Typical dose-response curves are often fitted to a four-parameter sigmoidal [25], such as:

$$\begin{array}{c} R\left(L\right)=\frac{A-D}{1+{\left(L/EC_{50}\right)}^{B}}+D \end{array}$$ (9)

where the physiological response *R*(*L*) for a given drug concentration *L* is characterized by its maximal *D* and minimal *A* asymptotes, B is the slope parameter of the curve, and *EC*_50_ is the half-maximal effective concentration of the ligand, that is, the inflection point of the curve. S1A Fig. shows a schematic representation of the workflow used to solve the model equations and obtain the dynamics of growth of the heterogeneous cell population.

As a numerical solution, the model is informed with data from a synthetically designed chimeric ligand composed of the Epidermal Growth Factor (EGF) as TE and different mutants of Interferon alfa-2a (IFN*α*-2a) as AE [15]. Thus, the apoptotic effect triggered by IFN*α*-2a stimulation is directed towards cells overexpressing the Epidermal Growth Factor Receptor (EGFR). The physiological response of the cells to the treatment corresponds to the apoptotic effect induced by IFN*α*-2a, measured experimentally as the percentage of surviving cells after 60 hours of treatment.

Since EGFR is an oncogene overexpressed in a number of tumor cells [28], this chimera can be potentially used to selectively target cancer cells without affecting the healthy surrounding tissue. Different mutants of the IFN*α*-2a molecule are tested as monomers (*M*_*wt*_, *M*_1_, *M*_2_ and *M*_3_), and as AE’s in chimeric configuration, identified here as *Ch*_*wt*_ for the chimera composed of the wild type version of the IFN*α*-2a linked to EGF, and *Ch*_1_, *Ch*_2_ for the experimentally available mutants of IFN*α*-2a with reduced affinity towards the IFN*α*-2a receptor linked to the targeting element EGF [15]. Other potential chimeras composed of EGF linked to IFN*α*-2a mutants with decreasing affinity towards the AER combined with EGF, named *Ch*_3_ and *Ch*_4_, are included in the analysis (Table 1 shows the dissociation constants for each IFN monomer).

**Table 1:** Parameters used in the population model. Values of IFN*α*-2a (AE) dissociation rates (*k*_*D*_), corresponding to IFN*α*-2a-IFNR wild type and mutants of IFN*α*-2a, from recent publications [15, 39] and theoretical ligands (*M*_3_ and *M*_3_). Mean values of EGF and IFN receptors expressed by Daudi and Daudi-EGFR cells, extracted from [15].

Parameter	Value	Units	Reference
*k*_*D*_ *M*_*wt*_	3	*nM*	Ref. [39]
*k*_*D*_ *M*_1_—K133A	26	*nM*	Ref. [39]
*k*_*D*_ *M*_2_—R144A	120	*nM*	Ref. [39]
*k*_*D*_ *M*_3_	240	*nM*	theoretical
*k*_*D*_ *M*_4_	480	*nM*	theoretical
*k*_*D*_ *EGF*	2.47	*nM*	theoretical
[*EGFR*]_*healthy*_	22	*molecules*	Ref. [15]
[*IFNR*]_*healthy*_	2800	*molecules*	Ref. [15]
[*EGFR*]_*unhealthy*_	5640	*molecules*	Ref. [15]
[*IFNR*]_*unhealthy*_	3600	*molecules*	Ref. [15]
# of cells at t = 0	100	*cells*	theoretical
Average cell cycle	27.5	*hours*	Ref. [29, 40]
Total time	60	*hours*	theoretical

To mimic the experimental conditions, cells in the population are allowed to proliferate for 60 hours in the presence of the drug treatment, and the physiological response of each cell to the treatment depends on the amount and efficiency of each ligand, the amount of TER and AER receptors expressed, and the exposure time to treatment. Given that the physiological response of the cells is apoptosis, we set the decision between survival or death for each cell in the population as follows: for a given physiological response (0 < *R*(*L*) < 1), the probability of undergoing apoptosis at every time point is computed as *θ* = (1 − *R*(*L*))^Δ*t*/*T*^, being *T* the total length of the experiment and Δt the time step in the simulation. Then, a random number (0 < *γ* < 1) from a uniform distribution is assigned for each cell in the population and compared to the value of *θ* at every time step. If *γ* ≥ *θ*, the cell survives. On the contrary, if *γ* < *θ*, then the cell dies and it is no longer considered in the simulation.

A cell division occurs when the age of a given cell reaches the numerical value for the cell cycle length assigned to that particular cell. This value is obtained from a gamma distribution with mean m = 27.5 hours [29, 30] and standard deviation of 2 hours. The amount of surface receptors are also gamma distributed [31], with a mean value obtained from experimental data [15] (see Table 1) and a coefficient of variation of 0.3, to mimic cell-to-cell variability in both populations. Mean values of the final cell numbers are obtained from 10 independent runs of the model. Numerical solution of the model equations and other calculations are performed using a in-house Matlab script (code available upon request).

## Results

### Selectivity of chimeric drugs versus monomers in a cell population

**Fig 2:**
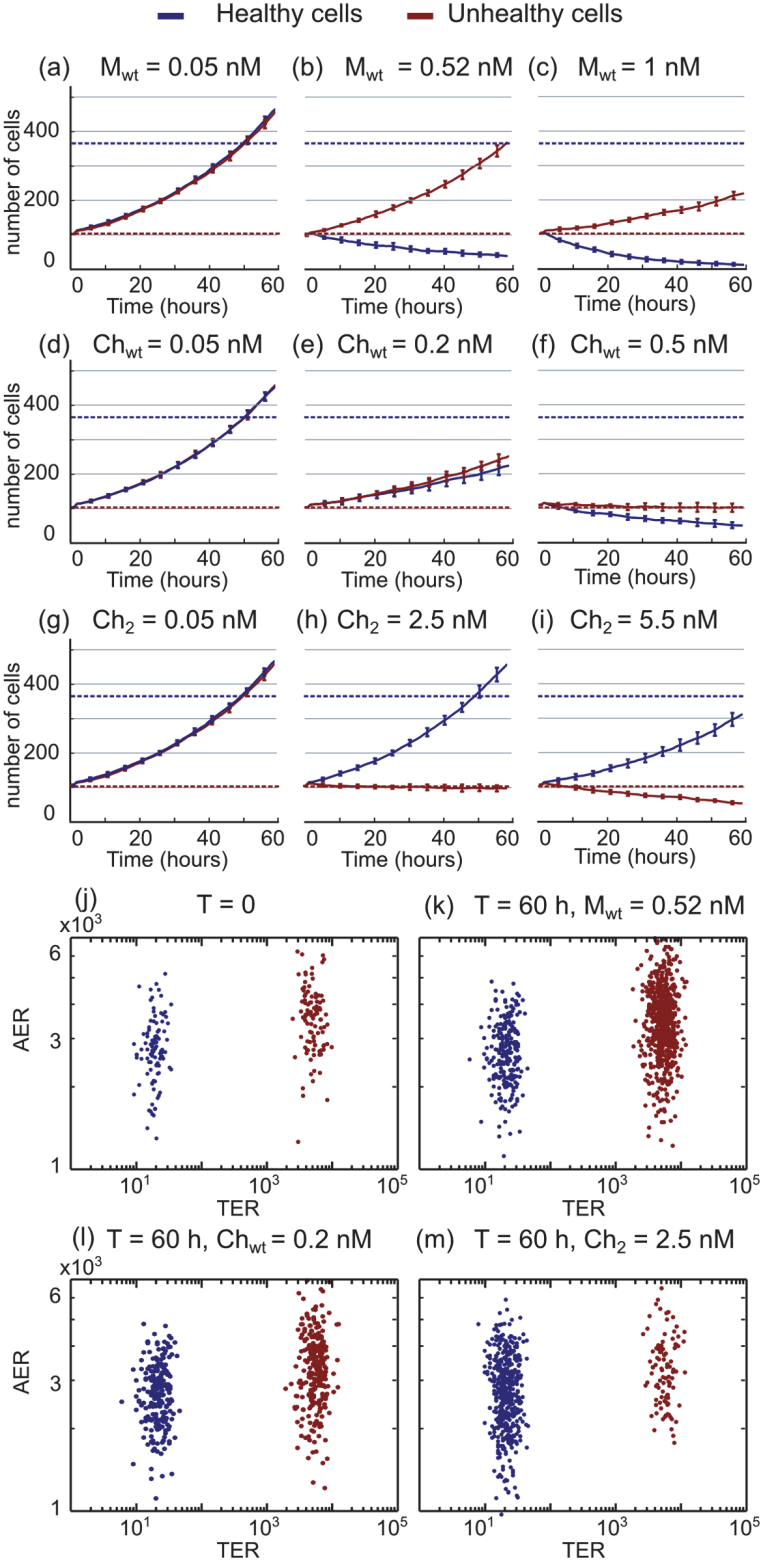
Dynamics of the cell populations after individual drug treatment. (A–C) Numerical solution of the model equations showing the time evolution of healthy (blue line) and unhealthy (red line) cells after treatment with low, intermediate and high concentrations of the *M*_*wt*_ monomer. (D–F) Time evolution after treatment with different concentrations of *Ch*_*wt*_. (G–I) Time evolution after treatment with different concentrations of *Ch*_2_, which at intermediate values is able to achieve the threshold of 80% survival of healthy cells, while the unhealthy cells are maintained. Solid line and error bars correspond to the average and standard deviation of 10 independent runs of the model. (J–M) Distribution of the AER and TER receptors for the two cell populations at (J) initial conditions, and after 60 hours of treatment with (K) *M*_*wt*_ = 0.52 nM, (E) *Ch*_*wt*_ = 0.2 nM and (M) *Ch*_2_ = 2.5 nM (i.e, same concentrations of Fig. 2B,E,H, respectively) Each dot corresponds to a cell in the population. Notice that *M*_*wt*_ treatment affects mainly the unhealthy cells (blue line, B,C), while *Ch*_2_ treatment shows a greater effect on the unhealthy population (red line, H–I).

The model described above is used to illustrate the effect of monomers versus chimeric ligands in a heterogeneous cell population. Fig. 2 illustrates the dynamics of growth of healthy (blue curve) and unhealthy (red curve) cell populations under nonselective monomers versus selective chimeric ligand treatment. To characterize and compare its selective potential, we define a threshold based on the amount of cells of both populations that remain after 60 hours of treatment (i.e., the duration of the experiments in [15], where the dose-response curves and other experimental data are obtained). Thus, a given treatment is considered as efficient when the number of unhealthy cells does not increase, while the population of healthy cells grows to at least 80% of its potential size. These two threshold values are marked in Fig. 2A–I as dashed red and blue horizontal lines for the unhealthy and healthy cells, respectively. These threshold values will be used to categorize the selective potential of a given treatment.

Low concentrations of *M*_*wt*_ are harmless to both cell populations, which can grow exponentially (Fig. 2A). Intermediate concentrations (Fig. 2B) have a much stronger effect in healthy cells than in unhealthy cells, due to higher resistance to IFN*α*-2a treatment in the unhealthy cell population (reported experimentally in [15], where authors hypothesized that this effect is mainly due to the anti-apoptotic potential of the EGFR overexpression [32] that may counteract the effect of IFN*α*-2a stimulation). Higher concentrations of the monomer are able to reduce the number of unhealthy cells in the system, but affecting the healthy population even more (Fig. 2C). This type of response is similar for all mutants of the monomer of IFN*α*-2a, as shown in the S2 Fig.

On the other hand, chimeric ligands show enhanced effect in cells overexpressing EGFR. The dynamics of *Ch*_*wt*_ ligand treatment, composed of EGF linked to wild type IFN*α*-2a, is shown in Fig. 2D–F. Low concentrations do not affect the growth rate of both cell populations. Intermediate and high values of chimeric ligand affect both healthy and unhealthy cells, reducing both cell populations simultaneously.

Optimal treatment can be achieved by the chimeric ligand composed of EGF linked to a mutant of IFN*α*-2a with reduced affinity towards the IFN receptor, as shown in Fig. 2G–I. Low concentrations of ligand *Ch*_2_ do not affect any of the populations, while intermediate values do allow the healthy cells to proliferate and prevent the unhealthy cell population to expand in size. Again, high concentrations of the ligand start to affect the healthy population that cannot grow above its 80% potential size. Dynamics for other chimeric ligands are plotted in S3 Fig.

Fig. 2J–M plots the amount of TER and AER for each cell of the healthy (blue) and unhealthy (red) population before treatment (Fig. 2J) and after 60 hours of treatment with intermediate concentrations of *M*_*wt*_ (Fig. 2K), *Ch*_*wt*_ (Fig. 2L) and *Ch*_2_ (Fig. 2M). Interestingly, despite the fact that the apoptotic potential of the monomers and chimeras directly depends on the amount of AER expressed by each individual cell, the model predicts that a number of cells with high values of AER does not undergo apoptosis after 60 hours of treatment. This is due to the fact that the physiological response of a given cell depends on the time that it has been under treatment and, since cells are continuously being born during the simulation, at t = 60 hours some recently born cells may not have been enough time under the influence of the drug to trigger apoptosis.

The above data evidences that selectivity in terms of the threshold of 80% can only be achieved using low affinity mutants of the AE subunit of the chimera, which are only efficient at very high concentrations of ligand (around 2 nM concentration for *Ch*_2_). This concentration representes a 4X increase compared to the concentration for *Ch*_*wt*_ = 0.5*nM*, which is the minimum concentration required to prevent the expansion of the unhealthy cell population at the expense of affecting strongly the healthy cells. This increase is also reported experimentally in [15], where the minimum concentration of *Ch*_2_ to prevent the unhealthy cell population to expand leaving the 80% of the healthy surrounding undamaged is 1.5nM, 3 times higher than the value of 0.5nM of *Ch*_*wt*_ that prevents the growth of the unhealthy cell population. Unfortunately, this higher doses of drug required to achieve selectivity can result in the emergence of other potential undesired effects, such as toxicity, or increased off-target interactions [33]. Therefore, strategies to reduce the total drug concentration for a given selective effect are relevant. In the next section, we show how combinations of selective chimeric ligands can reduce the concentration of total drug administered maintaining the selective potential.

### Synergistic interaction of selective chimeric drugs

**Fig 3:**
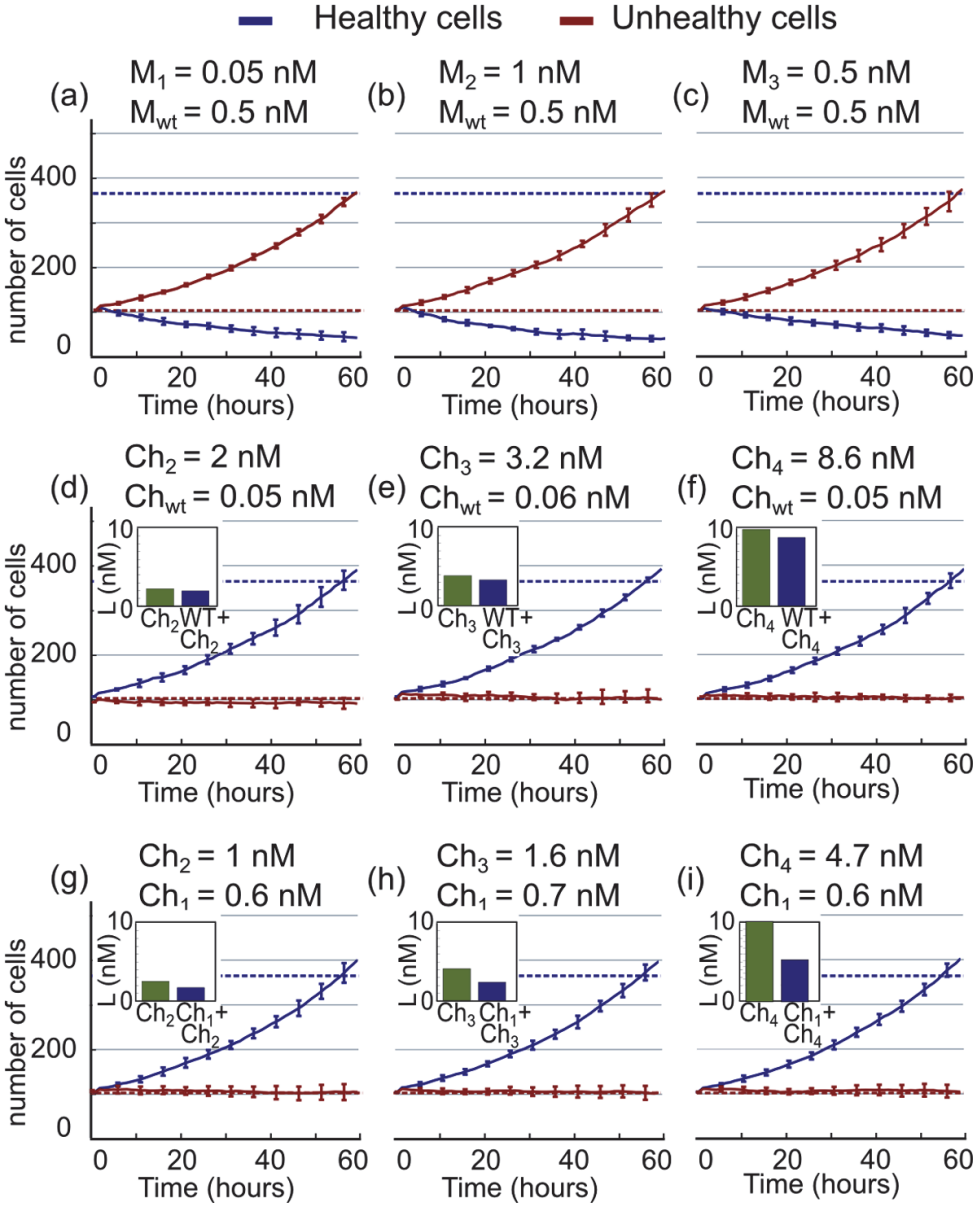
Synergistic performance of selective ligands. (A–C) Numerical solution of the model equations showing the time evolution of healthy (blue line) and unhealthy (red line) cells after treatment with different combinations of monomers, at the minimal concentration required to affect 20% of the unhealthy cell population. (D–F) Time evolution of different combinations of *Ch*_*wt*_ with other chimeric ligands at the minimal concentration required to achieve the threshold for selectivity. (G–I) Time evolution of different combinations of *Ch*_1_ with other chimeric ligands at the minimal concentration required to achieve the threshold for selectivity. Bars in each figure correspond to the minimal concentration for the threshold for single treatment and dual treatment with the chimeras used in each panel.

Mutant monomers of the same molecule act against the same target, therefore they behave as mutually exclusive and their interaction when combined is additive by definition. In this way, the lowest concentration to obtain a given effect always corresponds to the monomer with stronger binding affinity towards its receptor. Fig. 3A–C corresponds to simultaneous treatment of a fixed concentration of *M*_*wt*_ = 0.5nM with the minimal concentration of the mutants of IFN*α*-2a able to affect at least 20% of the unhealthy cells. According to their additive interaction, none of the potential combinations tested allows us to reduce the total drug concentration of IFN*α*-2a administered. In addition, none of the multiple combinations tested is able to mitigate the strong effect that the monomers exhibit towards the healthy cell population, as illustrated also in the next section.

Multi-drug treatment using selective drugs is shown by Fig. 3D–I, where we plot the dynamics of the two cell populations at the minimal concentration of total drug required to achieve the selectivity threshold for different combinations of the chimeras. Combinations of the poorly selective *Ch*_*wt*_ with chimeras *Ch*_2_, *Ch*_3_ and *Ch*_4_ are able to mitigate the expansion of the unhealthy cell population while meeting the 80% survival threshold of the healthy cells (Fig. 3D–F). The threshold is also achieved when combining the rest of the chimeras with *Ch*_1_, as shown in Fig. 3G–I.

Bars in each panel represent the minimal concentration of total drug to achieve the threshold of selectivity for each combination of ligands, compared to the same ligands as single treatment. We observe a slight reduction in the total concentration used for the combinatorial treatment compared to the individual treatment (values for the percentage of each reduction in the total concentration at threshold are listed in Table 2). Overall, the gain in performance of the dual treatment is more evident when combining a poorly selective ligand as *Ch*_1_ with a low affinity but highly selective chimera, such as *Ch*_3_. In this situation, we can achieve the threshold of optimal selectivity with a 50% reduction in the total drug concentration administered, compared to the concentration of *Ch*_3_ as individual treatment.

**Table 2:** Reduction in concentration at threshold for optimal selectivity. Percentage of reduction of total drug concentration of combinatorial treatment versus single drug treatment for different selective drug combinations.

Single drug	Drug combination	Reduction percentage
*Ch*_1_ = -	*Ch*_*wt*_+*Ch*_1_ = -	-%
*Ch*_2_ = 2.2	*Ch*_*wt*_+*Ch*_2_ = 2.0	7%
*Ch*_3_ = 4.2	*Ch*_*wt*_+*Ch*_3_ = 3.3	22%
*Ch*_4_ = 10.1	*Ch*_*wt*_+*Ch*_4_ = 8.6	14%
*Ch*_2_ = 2.4	*Ch*_1_+*Ch*_2_ = 1.6	33%
*Ch*_3_ = 4	*Ch*_1_+*Ch*_3_ = 2.3	42.5%
*Ch*_4_ = 10.1	*Ch*_1_+*Ch*_4_ = 5.3	47.5%

### The effect of combinatorial treatment can be estimated based on the effect of single treatment strategies

Detailed analysis of the drug interaction for each combination of two given ligands, as performed in the previous section, requires extensive computational resources. To overcome this, we use an additional approach to calculate the effect of a combination of drugs based on their effect when applied individually, using the following equation [22]:

$$\begin{array}{c} I=\frac{L_{c,1}}{L_{1}}+\frac{L_{c,2}}{L_{2}} \end{array}$$ (10)

where *L*_*c*,1_ and *L*_*c*,2_ correspond to the concentrations of the two drugs that produce a given effect when applied together, and *L*_1_ and *L*_2_ are the concentrations that induce the same effect when applied alone. The interaction index *I* depends on the type of interaction between the two drugs: synergistic (*I* < 1) additive (*I* = 1) or antagonistic (*I* > 1). In our particular case, since both ligands share common binding sites (i.e., they are mutants of the same molecule with reduced affinity), they behave as mutually exclusive and therefore, they follow the principle of Loewe additivity so the interaction index *I* in Eq. 10 is set to 1 [22] (a detailed analysis of the general equation for drug interaction in conditions of drug additivity, synergy or antagonism can be found as S1 Text).

Next, the Loewe additivity model is applied to drugs that induce a typical dose-response curve [25], as described in Eq. 9. Thus, we solve Eq. 9 for *L* and substitute into Eq. 10 for both ligands (*L* = *L*_1_ and *L* = *L*_2_), to obtain:

$$1=\frac{L_{c,1}}{EC_{50,1}}{\left(\frac{R(L_{c,1},L_{c,2})-D_{1}}{A_{1}-R(L_{c,1},L_{c,2})}\right)}^{\left(1/B_{1}\right)}+\frac{L_{c,2}}{EC_{50,2}}{\left(\frac{R(L_{c,1},L_{c,2})-D_{2}}{A_{2}-R(L_{c,1},L_{c,2})}\right)}^{\left(1/B_{2}\right)}$$ (11)

This equation can be solved numerically to obtain the physiological response *R*(*L*_*c*,1_, *L*_*c*,2_) for each potential combination of *L*_*c*,1_ and *L*_*c*,2_. The typical shape of the curve is shown in S4A Fig.

**Fig 4:**
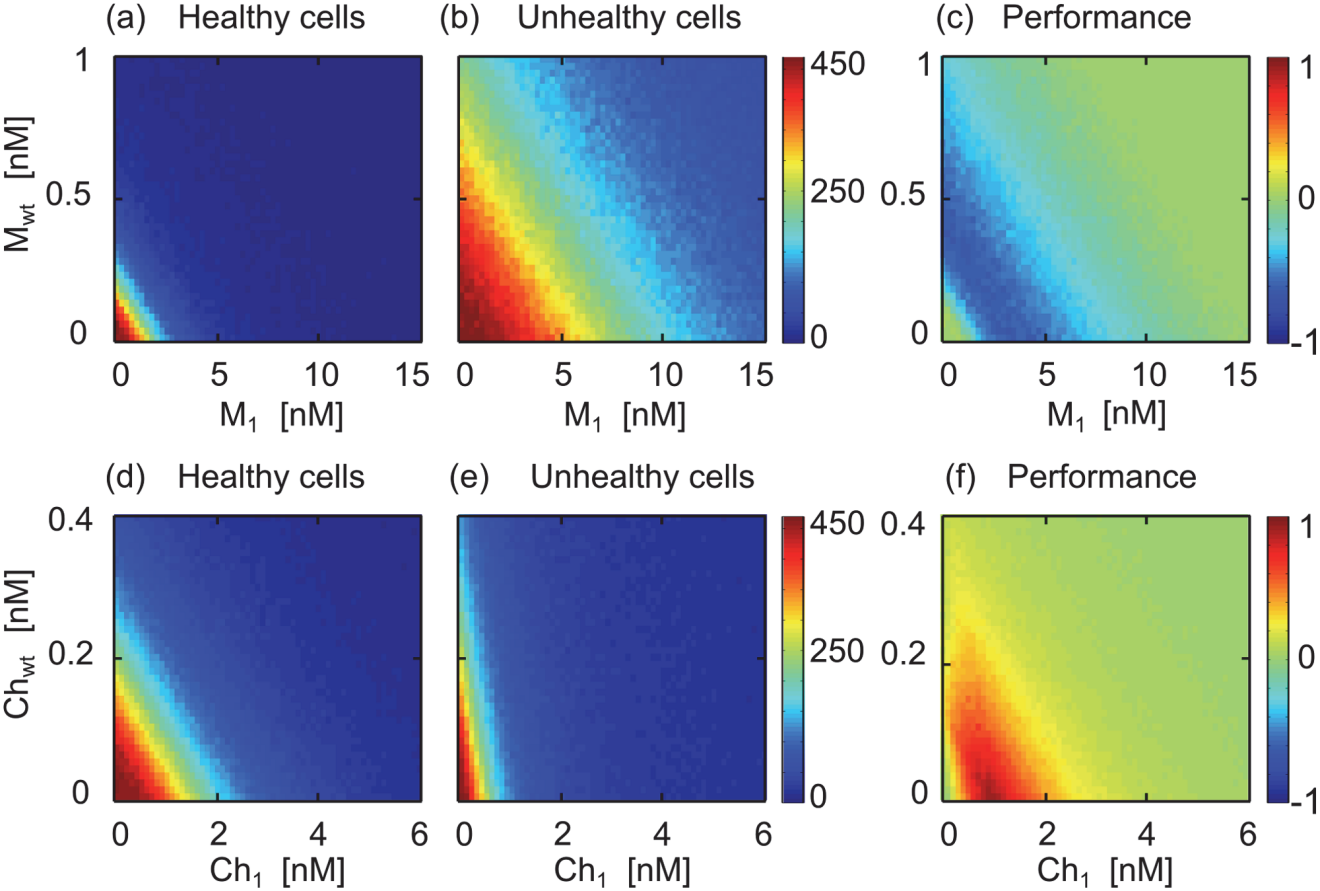
Isobolograms and performance colormaps for monomer and chimera combinations. (A–B) Isobolograms of the effect of the combination of monomers *M*_*wt*_ and *M*_1_ for (A) healthy and (B) unhealthy cells (final number of cells after 60 hours of treatment). (C) Performance of the combinatorial monomer treatment, where all values are below 0, evidencing that the combination affects more strongly the healthy population. (D–E) Isobolograms of the effect of chimeras *Ch*_*wt*_ + *Ch*_1_ for (D) healthy and (E) unhealthy cells. (F) Performance of the combinatorial chimeric treatment, where all values are above 0, evidencing that the combination affects more strongly the unhealthy population.

Therefore, Eq. 11 allows us to calculate directly the effect of the two drugs when applied simultaneously, significantly reducing the computational cost of the process. S5 Fig. plots the dynamics of several combinations of monomers and chimeras using this method (to be compared with Fig. 3, computed using the simultaneous drug stimulation of the system to show that both methods produce equivalent results). This simplified method allows us to compute the effect of any combination between two given drugs to develop isobolograms representing the final number of cells, after 60 hours of combined treatment for each combination of monomers (Fig. 4A–B) and chimeras (Fig. 4D–E).

Finally, to compare the selective effect of multiple combination of ligands, we define the performance *P*(*L*_*c*,1_, *L*_*c*,2_) of a given treatment as:

$$\begin{array}{c} P\left(L_{c,1},L_{c,2}\right)=N_{f}^{*}{\left(L_{c,1},L_{c,2}\right)}_{healthy}-N_{f}^{*}{\left(L_{c,1},L_{c,2}\right)}_{unhealthy} \end{array}$$ (12)

where $N_{f}^{*}{\left(L_{c,1},L_{c,2}\right)}_{healthy}$ and $N_{f}^{*}{\left(L_{c,1},L_{c,2}\right)}_{unhealthy}$ correspond to the final number *N*_*f*_ of healthy and unhealthy cells respectively, after 60 hours of combined treatment (i.e., the final point of the curves in Fig. 3) normalized to obtain values for the performance *P*(*L*_*c*,1_, *L*_*c*,2_) between −1 (minimal selectivity of treatment, i.e., 0% survival of the healthy cells) and 1 (optimal selectivity, i.e., ≥80% survival of healthy and no growth in the unhealthy cell population). A workflow scheme of this approach is shown in S1B Fig.

Fig. 4A–B illustrates the isobolograms for any concentration of monomers *M*_*wt*_ and *M*_1_ for healthy (Fig. 4A) and unhealthy cells (Fig. 4B) for a range of concentration values. The performance map (Fig. 4C) evidences that monomer combinations are not selective (i.e, *P* ≤ 0 at any concentration). Isobolograms for *Ch*_*wt*_ and *Ch*_1_ combinations are shown for healthy (Fig. 4D) and unhealthy (Fig. 4E) cell populations. The performance map (Fig. 4F) illustrates that the combination shows regions of positive performance (*P* > 0), i.e., regions where the combinatorial treatment acts selectively towards the unhealthy cell population.

**Fig 5:**
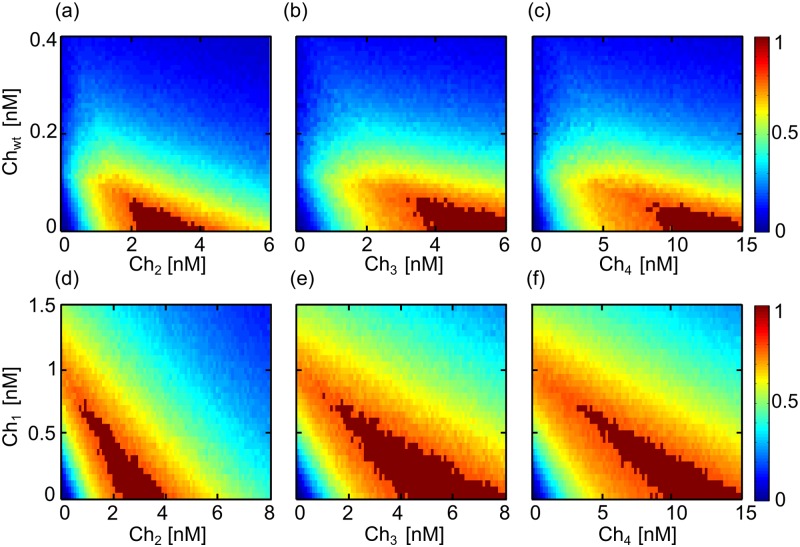
Performance colormaps for different chimera combinations. (A–C) Combination of *Ch*_*wt*_ with other chimeras. (D–F) Combination of *Ch*_1_ with other chimeras. Areas where the threshold of selectivity is achieved are marked in dark red. Since there is no negative values for the performance *P*, color bars are now presented between 0 and 1.

Performance colormaps for other combinations of chimeric drugs are shown in Fig. 5, where regions in which the threshold of selectivity is achieved are marked in dark red. Simultaneous treatment of *Ch*_*wt*_ with *Ch*_2_, *Ch*_3_ and *Ch*_4_ show that, for each combination, optimal selectivity can be achieved at slightly lower concentrations when using the two drugs simultaneously, compared to the same drugs acting alone (i.e., [*Ch*_*wt*_] = 0 in each panel). This is more evident when combining *Ch*_1_ with the other chimeras (Fig. 5D–F), where the optimal selectivity threshold is achieved at significantly lower concentrations using two selective drugs instead of one (numerical values for the minimal concentration for the threshold of selectivity as well as the reduction in total final concentration due to the combinatorial treatment are shown in Table 2).

### Synergistic interaction for selective chimeric drugs in cell populations with heritability

Previous simulations assumed that both healthy and unhealthy cells are, as a first approximation, phenotypically different, with the expression levels of TER and AER obtained from gamma distributions. Therefore, the amount of receptors expressed by a daughter cell depends on its cell type, but it is independent on the amount of receptors expressed by the mother cell. In other potential scenarios, the difference in phenotype between healthy and unhealthy cells can be caused by genetic mutations, and therefore, the amount of receptors expressed by the mother cell is inherited by the daughter cells. In these situations, a given treatment can become inefficient, and it can potentially act as selective pressure, acting more strongly over weak cells and ultimately inducing resistance to treatment in the population. This scenario has been explored extensively *in vivo* and *in silico*, and is one of the main causes of the short-lived response of targeted therapy in cancer [18]. Recently, it has been shown theoretically and experimentally that dual treatment strategies can dramatically reduce the possibility of development of resistant cells, resulting in long-term disease control compared to single drug treatment, or even sequential drug treatment [18].

**Fig 6:**
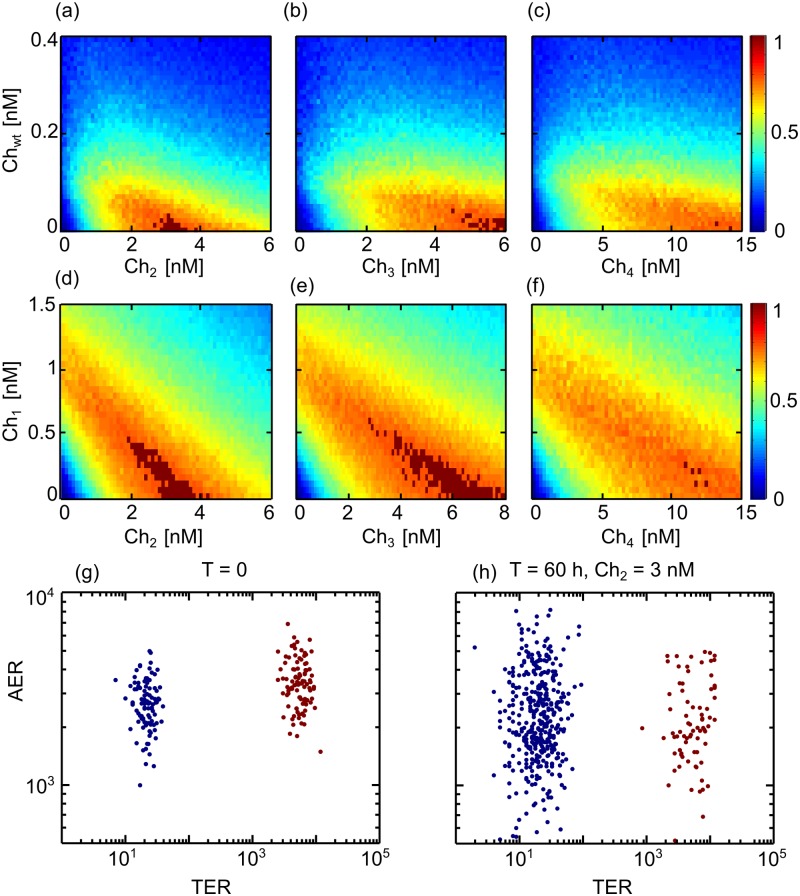
Performance colormaps for different chimera combinations in conditions of heritability. (A–C) Combination of *Ch*_*wt*_ with other chimeras. (D–F) Combination of *Ch*_1_ with other chimeras. Areas where the threshold of selectivity is achieved are marked in dark red. Since there is no negative values for the performance *P*, color bars are now presented between 0 and 1. (G–H) Distribution of the AER and TER receptors for the two cell populations before (G) and after (H) 60 hours of treatment with *Ch*_2_ = 3 nM.

To test the performance of dual selective treatment in the context of genetic inheritance of the amount of receptors, we set the average amount of TER and AER expressed by a given daughter cell as directly given by the amount of receptors expressed by the mother (with a coefficient of variation of 0.3). Simulations are performed as in the previous section, and performance colormaps can be computed for all possible concentrations of different ligand combinations (Fig. 6). Comparison of Fig. 6A–F with the corresponding Fig. 5A–F evidences that selectivity is more difficult to achieve in conditions of heritability (i.e., regions of optimal selectivity (marked in dark red) are reduced and occur at higher concentrations). Numerical values for the minimal concentration for the threshold of selectivity, as well as the reduction in total final concentration due to the combinatorial treatment in conditions of heritability, are shown in Table 3. Fig. 6 G–H, plots the values of AER and TER of the cells in the population before (Fig. 6G) and after (Fig. 6H) 60 hours of treatment for the minimal concentration of *Ch*_2_ that meets the threshold in conditions of heritability. Comparison of both distributions with conditions of no heritability (Fig. 2J,M) evidences that heritability increases the variability in the expression levels of AER and TER in the population, resulting in a decrease in the performance of the drug combinations and a reduction in the region of optimal selectivity (Fig. 6A–F).

**Table 3:** Reduction in concentration at threshold for optimal selectivity in conditions of heritability. Percentage of reduction of total drug concentration of combinatorial treatment versus single drug treatment for different selective drug combinations.

Single drug	Drug combination	Reduction percentage
*Ch*_2_ = 2.9	*Ch*_*wt*_+*Ch*_2_ = -	-%
*Ch*_3_ = 5.3	*Ch*_*wt*_+*Ch*_3_ = 4.5	15%
*Ch*_4_ = -	*Ch*_*wt*_+*Ch*_4_ = -	-%
*Ch*_2_ = 2.9	*Ch*_1_+*Ch*_2_ = 2.35	19%
*Ch*_3_ = 6	*Ch*_1_+*Ch*_3_ = 3.45	42.5%
*Ch*_4_ = -	*Ch*_1_+*Ch*_4_ = -	-%

## Conclusions and Discussion

Chimeric ligands with selective potential constitute one of the forefronts in modern pharmacology. The development of strategies to affect only malfunctioning cells inside a healthy tissue based on a sequential mechanisms of targeting is still in its early stages. Rational approaches based on modulating the strength of the interaction between ligand and target has shown that selectivity can be improved in a rational predictive manner [15, 25, 34]. Unfortunately, this results in a marked increase of the total concentration of drug that needs to be administered, which potentially increases the risk of toxicity and other undesired effects. Therefore, the problem of achieving selectivity at reduced drug concentrations is a main concern when developing selective drugs.

Our previous modeling approaches [25, 34] allow us to predict the optimal value of the affinity and dissociation rates of both AE and TE for improved selectivity at the lowest drug concentration. Unfortunately, the affinity and dissociation rates in a given ligand-receptor interaction cannot be modulated gradually, since single mutations in the ligand change abruptly the binding and unbinding rates with the complementary receptor. In this sense, combination of two ligands can, in principle, result beneficial to improve the selective potential of the treatment since, for instance, highly potent ligands could affect cells expressing high TER concentrations, while more selective chimeras (i.e., with reduced potency in the AE subunit) could discriminate better between healthy and unhealthy levels of the target protein.

To our knowledge, our results constitute the first studies focused on the combination of selective drugs, by generalizing our previous results of single treatments with selective drugs [25] to study selective drug combinations in cell population models. Our studies show that the combination of selective drugs is synergistic in terms of their selective potential, i.e., the combination of selective drugs can reduce the total drug administered to achieve a given selective effect, compared to the same drugs acting alone. We also show that using a explicit model of two selective drugs is equivalent to a simplified model where the two drugs are assumed to interact additively. This alternative method allows us to develop performance maps where selectivity is computed for any given concentration of the combined drugs. We used a cell population based model to study how these types of treatments respond in a context of cell-variability and their robustness in condition where the amount of target proteins is inherited from mother to daughter cells. Interestingly, despite assuming additive interaction of the different chimeras when combined (i.e., they compete for the same molecular targets), when looking at the selective potential of the treatment, chimeras behave as synergistic.

Several main assumptions are taken into account while developing the model. First, we assume that production and degradation of each receptor is balanced in conditions of no ligand stimulation. We also simplified all potential downstream regulation in receptor expression after activation, focusing only on the regulation that takes place due to direct ligand stimulation. We also assume that the activity triggered by the ligand-receptor interaction is proportional to the amount of maximum active complexes formed. Other potential values such as the total value of active complexes at a given time also produce equivalent results, as discussed in [25] at the single cell level. Regarding the simulations of the population dynamics, we assumed that all cells proliferate at the same mean rate, independently of the amount of EGFR receptors. It is well-known that EGFR stimulation is correlated with the activation of proliferative signals [32], but experimental data monitoring differences in cell cycle length for Daudi versus Daudi-EGFR cells used to inform our model are not available [15]. In addition, the effect of heritability in the expression of receptors was assumed to simply depend on the amount of receptors expressed by the mother cells. Other potential scenarios to capture the effect of mutations in the regulation of the expression of receptors will be more realistic, but they will result in more free parameters and assumptions. In addition, we assume that the effect of heritability will be more relevant in longer experiments, i.e., when more generations of cells are allowed to develop. Unfortunately, experimental data are only available at the time point of t = 60h, corresponding to an average of 2.2 generations, insufficient to observe the selective pressure effect induced by the drug treatment. To mimic cell-to-cell variability in the population, we assumed gamma distributions for the amount of receptors expressed and the cell cycle length, based on several publications. Other types of distributions were also tested (gaussian, lognormal), with almost no difference in the results compared to the gamma distribution [35–38]. To quantitatively compare the different combinatorial treatments, a threshold is defined in terms of the potential selectivity of the treatment towards the different cell types expressing different concentrations of the target proteins (80% survival of the healthy cells while the number of unhealthy cells is maintained). Other potential threshold values defined also evidence the reported synergism when combining two selective ligands, but at different drug concentrations.

In conclusion, we have shown that combination of selective drugs can selectively affect a given cell population at reduced concentrations compared to single drug treatment. These types of theoretical studies focused on the rational design of selective drugs and treatments can complement experimental efforts, allowing researches to develop a more reliable and efficient approach to quantitative pharmacology.

## Supporting Information

S1 Text Response surface plots for drug interaction in conditions of drug additivity, synergy or antagonism. (TEX)

S1 Fig Workflow to obtain the effect of a drug combination on a cell population. (A) Direct simulation of two simultaneous treatments (see section Models: Eqs. 1–4 are solved directly for two simultaneous ligands (*j* = 1,2) at a constant concentration. The value of AER-AE complexes formed is then translated into a physiological effect using calibration with experimental dose response curves obtained from [15]. Two populations of cells are defined with values for AER and TER from gamma distributions for healthy and unhealthy cells. Eqs. 1–4 are solved numerically for each cell in the two populations, obtaining the dynamics of growth for healthy and unhealthy cell populations for a given constant concentration of *L*_1_ and *L*_2_. (B) Calculation of the effect of combinatorial treatment assuming additive interaction between ligands: the maximum number of AER-AE complexes is calculated for each combination of AER and TER receptors concentrations by solving Eqs. 1–4 for a single ligand treatment (*j* = 1). The output of the model is translated to a calibration curve [25], obtaining the theoretical dose-response curves for each ligand. Physiological response curves are fitted to a four-parameter sigmoidal (Eq.9), and the physiological response for any concentration of two ligands is then calculated using the Loewe approximation for additive ligand interaction (Eq. 11). This response is then used to perform simulations for healthy and unhealthy cell populations, following the same procedure as in (A). Finally, the number of healthy and unhealthy cells after 60 hours of treatment is plotted in the corresponding isobologram for each ligand combination. The final performance colormap for each value of the combination of ligands is obtained by subtracting the normalized isobolograms for unhealthy minus healthy cells. Values above threshold of performance are highlighted in dark red.
(TIFF)

S2 Fig Dynamics of the cell populations after individual monomer treatment. Numerical solution of the model equations showing the time evolution of healthy (blue line) and unhealthy (red line) cells after treatment with low, intermediate and high concentrations of (A–C) *M*_1_ monomer, (D–F) *M*_2_ monomer, and (G–I) *M*_3_ monomer.
(TIFF)

S3 Fig Dynamics of the cell populations after individual chimeric treatment. Numerical solution of the model equations showing the time evolution of healthy (blue line) and unhealthy (red line) cells after treatment with low, intermediate and high concentrations of (A–C) *Ch*_1_ chimera, (D–F) *Ch*_3_ chimera, and (G–I) *Ch*_4_ chimera.
(TIFF)

S4 Fig Response surfaces for *L*_1_ + *L*_2_ combinations. Response surface plots using Eq.13 (see S1 Text) for two ligands showing (A) additivity, *α* = 0, (B) synergy, *α* = 5 and (C) antagonism, *α* = −0.5. The black curve is the isobol curve (i.e., curve of equal effect) for 50% (*EC*_50_) of physiological response and it has different curvature depending on the interaction type.
(TIFF)

S5 Fig Numerical solution of the model equations showing synergistic performance of selective ligands using the Loewe approximation (to be compared with Fig. 3 in the main text, obtained using chemical dynamics simulation of two ligands simultaneously). (A–C) Time evolution of healthy (blue line) and unhealthy (red line) cells after treatment with different combinations of monomers, at the minimal concentration required to affect 20% of the unhealthy cell population. (D–F) Time evolution of different combinations of *Ch*_*wt*_ with other chimeric ligands at the minimal concentration required to achieve the threshold for selectivity. (G–I) Time evolution of different combinations of *Ch*_1_ with other chimeric ligands at the minimal concentration required to achieve the threshold for selectivity.
(TIFF)

## References

[1] Croce CM (2008) Oncogenes and cancer. New England Journal of Medicine 358: 502–511. 18234754 10.1056/NEJMra072367

[2] Verma S, Miles D, Gianni L, Krop IE, Welslau M, et al (2012) Trastuzumab Emtansine for HER2-Positive Advanced Breast Cancer. New England Journal of Medicine. 367(19), 1783–1791. 10.1056/NEJMoa1209124 23020162 PMC5125250

[3] Taylor ND, Way JC, Silver PA, Cironi P. (2010) Anti-glycophorin single-chain Fv fusion to low-affinity mutant erythropoietin improves red blood cell-lineage specificity. Protein Engineering, Design and Selection. 23(4):251–60. 10.1093/protein/gzp085 20083493

[4] Turturro F (2007) Denileukin diftitox: a biotherapeutic paradigm shift in the treatment of lymphoid-derived disorders. Expert Review of Anticancer Therapy 7: 11–17. 10.1586/14737140.7.1.11 17187516

[5] Kreitman RJ, Wilson WH, White JD, Stetler-Stevenson M, Jaffe ES, et al (2000) Phase i trial of recombinant immunotoxin anti-tac (fv)-pe38 (lmb-2) in patients with hematologic malignancies. Journal of Clinical Oncology 18: 1622–1636. 10764422 10.1200/JCO.2000.18.8.1622

[6] Kreitman RJ, Squires DR, Stetler-Stevenson M, Noel P, FitzGerald DJ, et al (2005) Phase i trial of recombinant immunotoxin rfb4 (dsfv)-pe38 (bl22) in patients with b-cell malignancies. Journal of clinical oncology 23: 6719–6729. 10.1200/JCO.2005.11.437 16061911

[7] Kioi M, Husain SR, Croteau D, Kunwar S, Puri RK (2006) Convection-enhanced delivery of interleukin-13 receptor-directed cytotoxin for malignant glioma therapy. Technology in cancer research & treatment 5: 239–250. 10.1177/153303460600500307 16700620

[8] Bremer E, Samplonius DF, van Genne L, Dijkstra MH, Kroesen BJ, et al (2005) Simultaneous inhibition of epidermal growth factor receptor (egfr) signaling and enhanced activation of tumor necrosis factor-related apoptosis-inducing ligand (trail) receptor-mediated apoptosis induction by an scfv: strail fusion protein with specificity for human egfr. Journal of Biological Chemistry 280: 10025–10033. 15644326 10.1074/jbc.M413673200

[9] Bremer E, Samplonius DF, Peipp M, van Genne L, Kroesen BJJ, et al (2005) Target cell–restricted apoptosis induction of acute leukemic t cells by a recombinant tumor necrosis factor–related apoptosis-inducing ligand fusion protein with specificity for human cd7. Cancer research 65: 3380–3388. 15833872 10.1158/0008-5472.CAN-04-2756

[10] Stieglmaier J, Bremer E, Kellner C, Liebig TM, ten Cate B, et al (2008) Selective induction of apoptosis in leukemic b-lymphoid cells by a cd19-specific trail fusion protein. Cancer Immunology, Immunotherapy 57: 233–246. 10.1007/s00262-007-0370-8 17665197 PMC11030665

[11] Bremer E, ten Cate B, Samplonius DF, de Leij LF, Helfrich W (2006) Cd7-restricted activation of fas-mediated apoptosis: a novel therapeutic approach for acute t-cell leukemia. Blood 107: 2863–2870. 10.1182/blood-2005-07-2929 16332967

[12] Bremer E, ten Cate B, Samplonius DF, Mueller N, Wajant H, et al (2008) Superior activity of fusion protein scfvrit: sfasl over cotreatment with rituximab and fas agonists. Cancer research 68: 597–604. 10.1158/0008-5472.CAN-07-5171 18199557

[13] Xuan C, Steward KK, Timmerman JM, Morrison SL (2010) Targeted delivery of interferon-alpha via fusion to anti-cd20 results in potent antitumor activity against b-cell lymphoma. Blood 115: 2864–2871. 10.1182/blood-2009-10-250555 20139095 PMC2854431

[14] Zhang B, Gao B, Dong S, Zhang Y, Wu Y (2011) Anti-tumor efficacy and pre-clinical immuno-genicity of ifnα2a-ngr. Regulatory Toxicology and Pharmacology 60: 73–78. 10.1016/j.yrtph.2011.02.007 21338646

[15] Cironi P, Swinburne IA, Silver PA (2008) Enhancement of cell type specificity by quantitative modulation of a chimeric ligand. Journal of biological chemistry 283: 8469–8476. 10.1074/jbc.M708502200 18230610 PMC2417160

[16] Fitzgerald JB, Schoeberl B, Nielsen UB, Sorger PK (2006) Systems biology and combination therapy in the quest for clinical efficacy. Nature chemical biology 2: 458–466. 10.1038/nchembio817 16921358

[17] Yuan S, Wang F, Chen G, Zhang H, Feng L, et al (2013) Effective elimination of cancer stem cells by a novel drug combination strategy. Stem Cells 31: 23–34. 10.1002/stem.1273 23132831 PMC3538380

[18] Bozic I, Reiter JG, Allen B, Antal T, Chatterjee K, et al (2013) Evolutionary dynamics of cancer in response to targeted combination therapy. Elife 2 10.7554/eLife.00747 23805382 PMC3691570

[19] Tan X, Hu L, Luquette LJ III, Gao G, Liu Y, et al (2012) Systematic identification of synergistic drug pairs targeting hiv. Nature biotechnology 30: 1125–1130. 10.1038/nbt.2391 23064238 PMC3494743

[20] Jia J, Zhu F, Ma X, Cao ZW, Li YX, et al (2009) Mechanisms of drug combinations: interaction and network perspectives. Nature reviews Drug discovery 8: 111–128. 10.1038/nrd2683 19180105

[21] Borisy AA, Elliott PJ, Hurst NW, Lee MS, Lehár J, et al (2003) Systematic discovery of multi-component therapeutics. Proceedings of the National Academy of Sciences 100: 7977–7982. 10.1073/pnas.1337088100 PMC164698 12799470

[22] Berenbaum MC (1977) Synergy, additivism and antagonism in immunosuppression. a critical review. Clinical and experimental immunology 28: 1. 324671 PMC1540873

[23] Zimmermann GR, Lehar J, Keith CT. (2007) Multi-target therapeutics: when the whole is greater than the sum of the parts. Drug Discovery Today 12: 34–42 10.1016/j.drudis.2006.11.008 17198971

[24] Lehaŕ J, Krueger AS, Avery W, Heilbut AM, Johansen LM, et al (2009) Synergistic drug combinations tend to improve therapeutically relevant selectivity. Nature Biotechnology 27, 659–666 10.1038/nbt.1549 19581876 PMC2708317

[25] Doldán-Martelli V, Guantes R, Miguez DG (2013) A mathematical model for the rational design of chimeric ligands in selective drug therapies. CPT: Pharmacometrics & Systems Pharmacology 2: e26. 23887616 10.1038/psp.2013.2 PMC3600755

[26] Lauffenburger DA, Linderman JJ (1993) Receptors: models for binding, trafficking, and signaling, volume 365 Oxford University Press New York:.

[27] Míguez DG (2010) The role of asymmetric binding in ligand–receptor systems with 1: 2 interaction ratio. Biophysical chemistry 148: 74–81. 10.1016/j.bpc.2010.02.012 20332059

[28] Arteaga CL (2001) The epidermal growth factor receptor: from mutant oncogene in nonhuman cancers to therapeutic target in human neoplasia. Journal of Clinical Oncology 19: 32s–40s. 11560969

[29] Gewert DR, Shah S, Clemens MJ (1981) Inhibition of cell division by interferons. European Journal of Biochemistry 116: 487–492. 10.1111/j.1432-1033.1981.tb05362.x 6167441

[30] Klein E, Klein G, Nadkarni JS, Nadkarni JJ, Wigzell H, et al (1968) Surface igm-kappa specificity on a burkitt lymphoma cell in vivo and in derived culture lines. Cancer Research 28: 1300–1310. 4174339

[31] Cai L, Friedman N, Xie XS (2006) Stochastic protein expression in individual cells at the single molecule level. Nature 440: 358–362. 10.1038/nature04599 16541077

[32] Oda K, Matsuoka Y, Funahashi A, Kitano H (2005) A comprehensive pathway map of epidermal growth factor receptor signaling. Molecular systems biology 1 10.1038/msb4100014 16729045 PMC1681468

[33] MacDonald ML, Lamerdin J, Owens S, Keon BH, Bilter GK, et al (2006) Identifying off-target effects and hidden phenotypes of drugs in human cells. Nature Chemical Biology 2: 329–337. 10.1038/nchembio790 16680159

[34] Ruiz-Herrero T, Estrada J, Guantes R, Miguez DG (2013) A tunable coarse-grained model for ligand-receptor interaction. PLoS computational biology 9: e1003274 10.1371/journal.pcbi.1003274 24244115 PMC3828130

[35] Weddell JC, Imoukhuede PI (2014) Quantitative characterization of cellular membrane-receptor heterogeneity through statistical and computational modeling. PloS one 9: e97271 10.1371/journal.pone.0097271 24827582 PMC4020774

[36] Bengtsson M, Stahlberg A, Rorsman P, Kubista M (2005) Gene expression profiling in single cells from the pancreatic islets of langerhans reveals lognormal distribution of mrna levels. Genome research 15: 1388–1392. 10.1101/gr.3820805 16204192 PMC1240081

[37] Dowling MR, Milutinović D, Hodgkin PD (2005) Modelling cell lifespan and proliferation: is like-lihood to die or to divide independent of age? Journal of The Royal Society Interface 2: 517–526. 10.1098/rsif.2005.0069 PMC1618504 16849210

[38] Friedman N, Cai L, Xie XS (2006) Linking stochastic dynamics to population distribution: An analytical framework of gene expression. Phys Rev Lett 97: 168302 10.1103/PhysRevLett.97.168302 17155441

[39] Piehler J, Roisman LC, Schreiber G (2000) New structural and functional aspects of the type i interferon-receptor interaction revealed by comprehensive mutational analysis of the binding interface. Journal of Biological Chemistry 275: 40425–40433. 10.1074/jbc.M006854200 10984492

[40] Schwarz MA, Tardelli L, Macosko HD, Sullivan LM, Narula SK, et al (1995) Interleukin 4 retards dissemination of a human b-cell lymphoma in severe combined immunodeficient mice. Cancer research 55: 3692–3696. 7641177

