# Supplementary figures and images for: Synergistic Interaction between Selective Drugs in Cell Populations Models
Source: PLoS One. 2015 Feb 11;10(2):e0117558. doi: 10.1371/journal.pone.0117558 (PMC4324767; doi:10.1371/journal.pone.0117558)

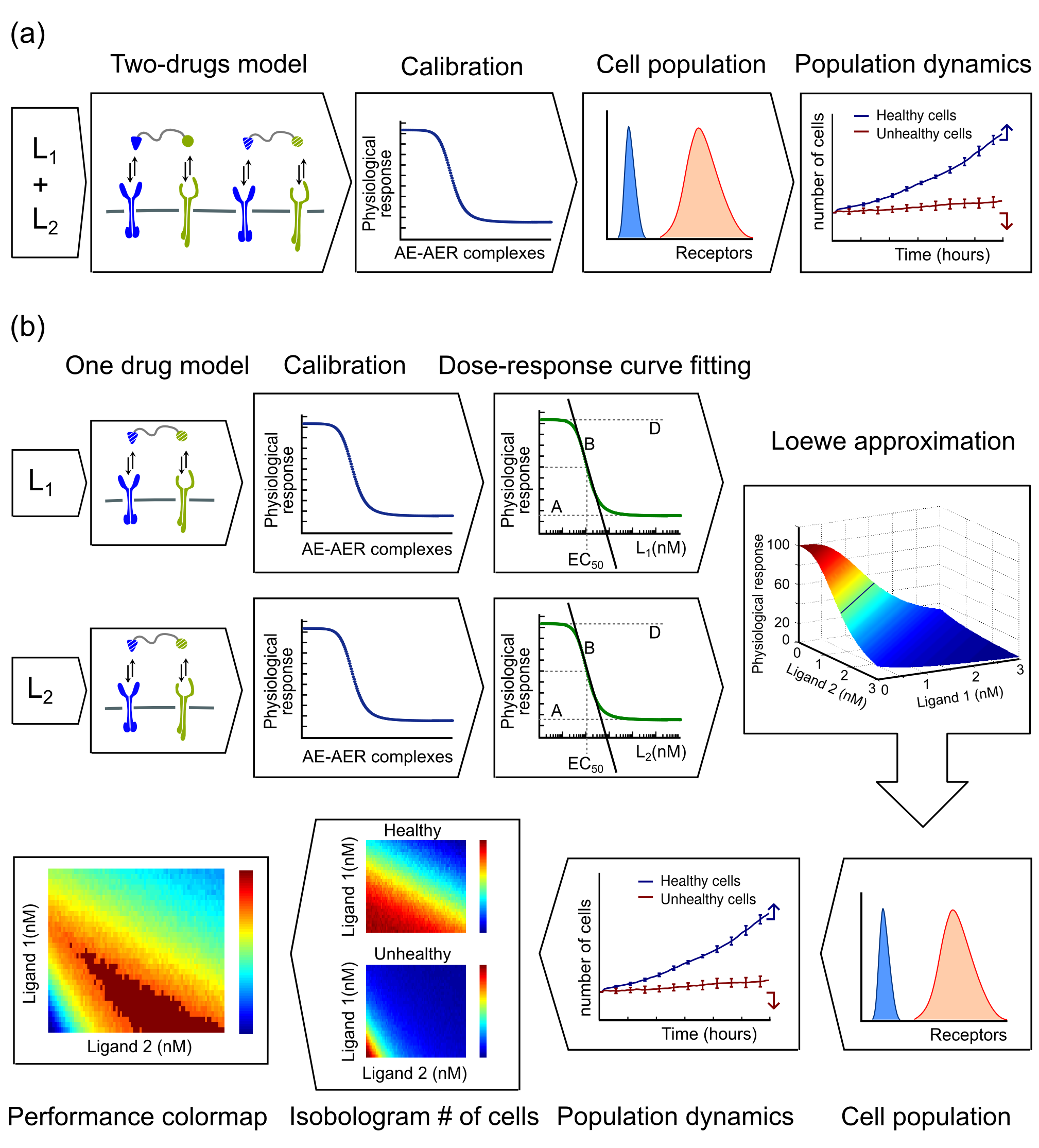

Supplement: S1 Fig — (A) Direct simulation of two simultaneous treatments (see section Models: Eqs. 1–4 are solved directly for two simultaneous ligands (j = 1,2) at a constant concentration. The value of AER-AE complexes formed is then translated into a physiological effect using calibration with experimental dose response curves obtained from [15]. Two populations of cells are defined with values for AER and TER from gamma distributions for healthy and unhealthy cells. Eqs. 1–4 are solved numerically for each cell in the two populations, obtaining the dynamics of growth for healthy and unhealthy cell populations for a given constant concentration of L 1 and L 2. (B) Calculation of the effect of combinatorial treatment assuming additive interaction between ligands: the maximum number of AER-AE complexes is calculated for each combination of AER and TER receptors concentrations by solving Eqs. 1–4 for a single ligand treatment (j = 1). The output of the model is translated to a calibration curve [25], obtaining the theoretical dose-response curves for each ligand. Physiological response curves are fitted to a four-parameter sigmoidal (Eq.9), and the physiological response for any concentration of two ligands is then calculated using the Loewe approximation for additive ligand interaction (Eq. 11). This response is then used to perform simulations for healthy and unhealthy cell populations, following the same procedure as in (A). Finally, the number of healthy and unhealthy cells after 60 hours of treatment is plotted in the corresponding isobologram for each ligand combination. The final performance colormap for each value of the combination of ligands is obtained by subtracting the normalized isobolograms for unhealthy minus healthy cells. Values above threshold of performance are highlighted in dark red. (TIFF) [file pone.0117558.s002.tiff]

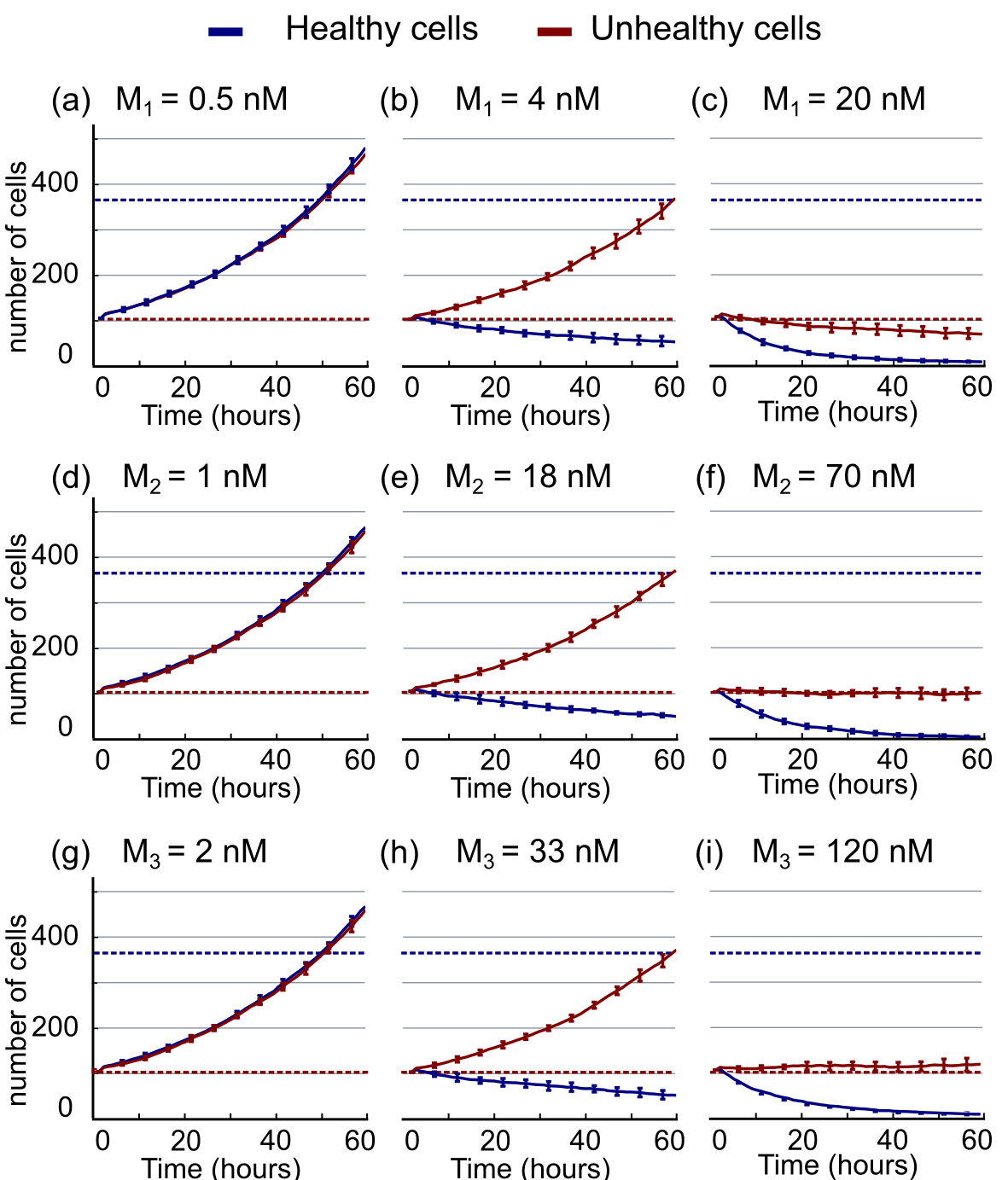

Supplement: S2 Fig — Numerical solution of the model equations showing the time evolution of healthy (blue line) and unhealthy (red line) cells after treatment with low, intermediate and high concentrations of (A–C) M 1 monomer, (D–F) M 2 monomer, and (G–I) M 3 monomer. (TIFF) [file pone.0117558.s003.tiff]

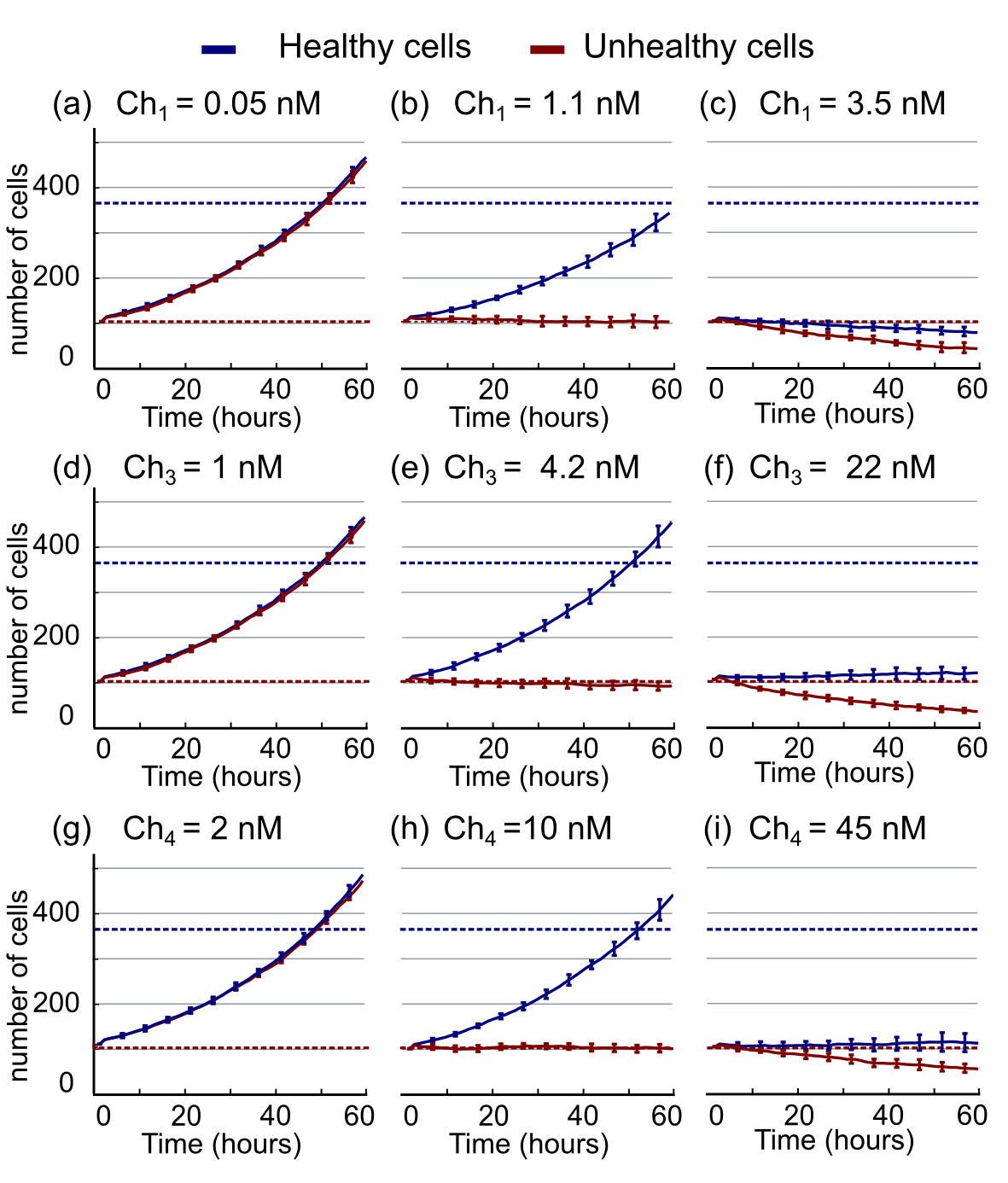

Supplement: S3 Fig — Numerical solution of the model equations showing the time evolution of healthy (blue line) and unhealthy (red line) cells after treatment with low, intermediate and high concentrations of (A–C) Ch 1 chimera, (D–F) Ch 3 chimera, and (G–I) Ch 4 chimera. (TIFF) [file pone.0117558.s004.tiff]

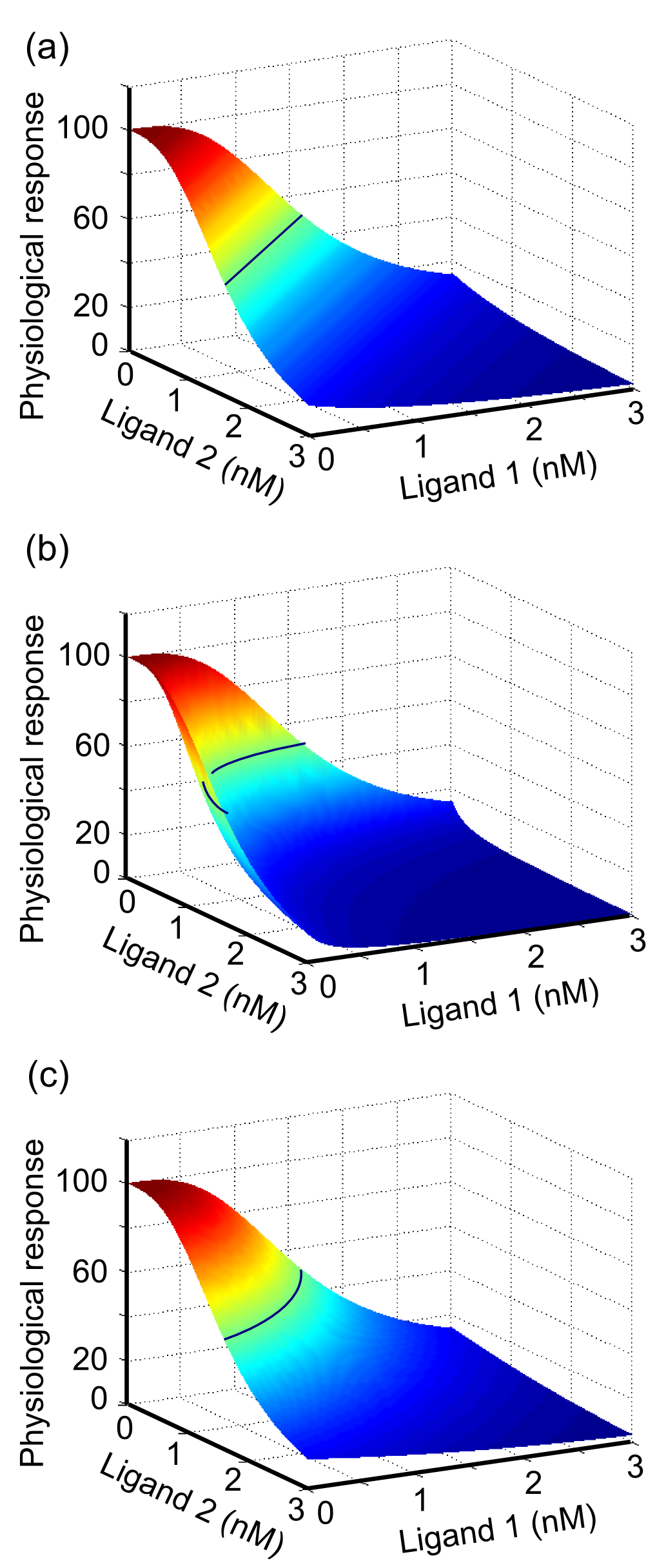

Supplement: S4 Fig — Response surface plots using Eq.13 (see S1 Text) for two ligands showing (A) additivity, α = 0, (B) synergy, α = 5 and (C) antagonism, α = −0.5. The black curve is the isobol curve (i.e., curve of equal effect) for 50% (EC 50) of physiological response and it has different curvature depending on the interaction type. (TIFF) [file pone.0117558.s005.tiff]

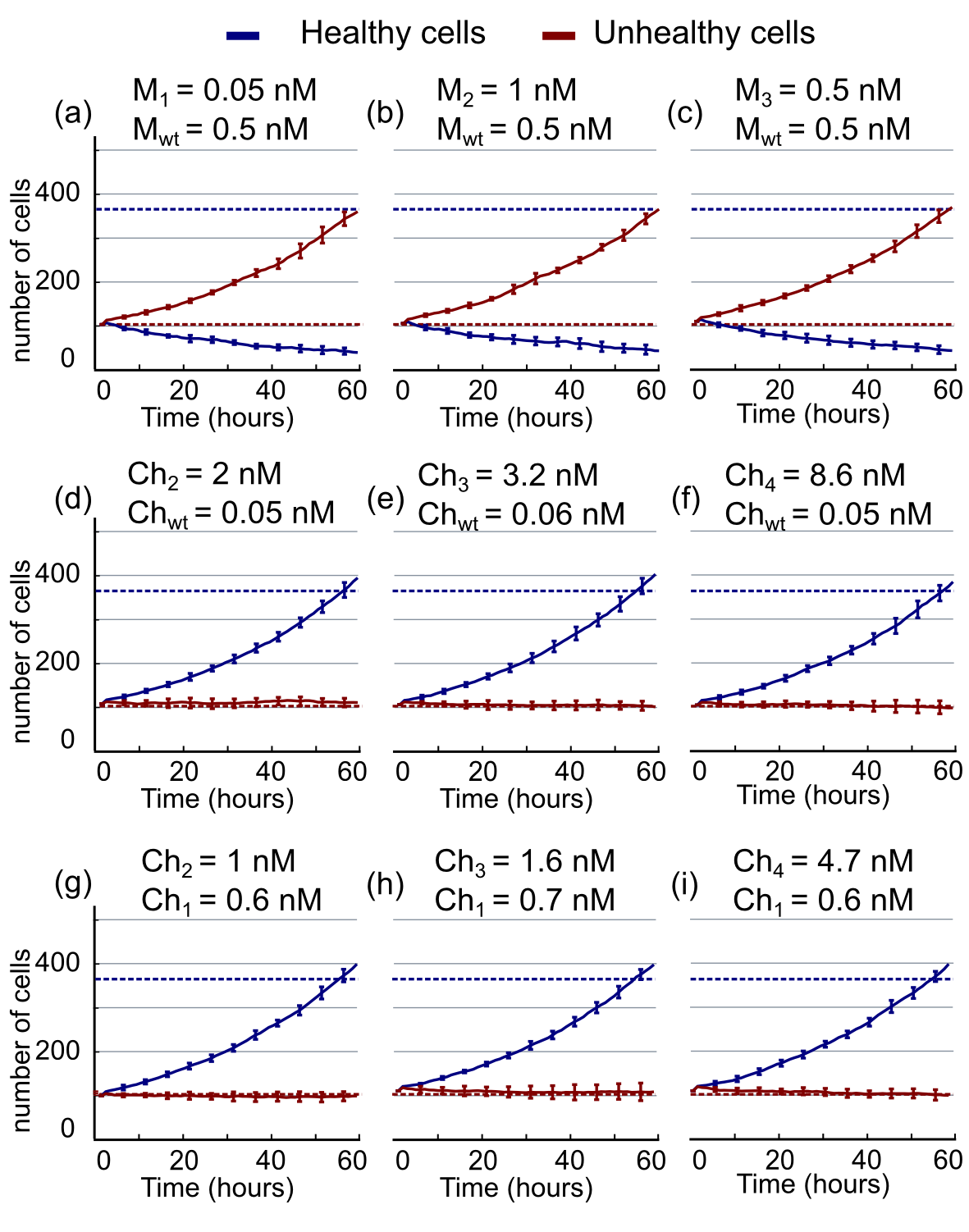

Supplement: S5 Fig — (A–C) Time evolution of healthy (blue line) and unhealthy (red line) cells after treatment with different combinations of monomers, at the minimal concentration required to affect 20% of the unhealthy cell population. (D–F) Time evolution of different combinations of Ch wt with other chimeric ligands at the minimal concentration required to achieve the threshold for selectivity. (G–I) Time evolution of different combinations of Ch 1 with other chimeric ligands at the minimal concentration required to achieve the threshold for selectivity. (TIFF) [file pone.0117558.s006.tiff]
